# Management of orthopedic plate exposure: a case series evaluating advanced wound care and delayed hardware removal

**DOI:** 10.1007/s00590-026-04700-6

**Published:** 2026-03-03

**Authors:** Riccardo Garibaldi, Paolo Ivan Fiore, Seraina Rietschi, Jerome Cottet, Olivia Zbinden, Angela Seidel

**Affiliations:** 1 HFR Fribourg Cantonal Hospital and University, 2/6 Chemin des Pensionnats, Villars-sur-Glâne, Switzerland; 2 Ente Ospedaliero Cantonale, Via Tesserete 46, Lugano, Switzerland

**Keywords:** Orthopedic implants, Plate exposure, Wound healing, Negative pressure wound therapy, Advanced wound dressings

## Abstract

**Background:**

Plate exposure following open reduction and internal fixation (ORIF) poses significant challenges, including infection risk and impaired fracture healing. Traditional management often involves aggressive surgical interventions.

**Methods:**

This retrospective case series examines seven patients treated between 2020 and 2025 who developed plate exposure after ORIF of lower extremity fractures. Management included local wound care with advanced dressings (Apligraf^®^, NuShield^®^), negative pressure wound therapy, targeted antibiotic therapy when indicated, and delayed hardware removal post-fracture consolidation.

**Results:**

All patients achieved complete wound healing, with a mean closure time of 143 days. Radiographic union was confirmed in all cases except one asymptomatic nonunion. Antibiotics were administered in four cases, guided by clinical signs and microbiological findings. No infections were detected at the time of hardware removal.

**Conclusion:**

A conservative approach utilizing advanced wound care techniques and delayed hardware removal can effectively manage plate exposure without necessitating early implant removal or flap coverage. This strategy may reduce patient morbidity and preserve fracture stability.

## Introduction

Wound dehiscence with subsequent exposure of orthopedic implants, especially after open reduction and internal fixation of fractures (ORIF), represents a significant challenge in orthopedic trauma surgery. The exposure of metallic implants increases the risk of deep infection and compromised fracture healing.

The risk of implant exposure is influenced by several factors, including the anatomical location, the quality of the overlying soft tissue, and the presence of comorbidities that impair wound healing [1]. Sites such as the distal tibia and calcaneus are particularly susceptible due to limited soft tissue coverage and poor vascularity [1]. When wound dehiscence leads to implant exposure the treatment goal is to eradicate the infection while maintaining mechanical stability of the fracture [2].

Traditional management strategies have often emphasized aggressive surgical debridement with early implant removal and soft tissue reconstruction using local or free flaps. Although the use of coverage flaps and skin grafts remains a valid strategy, they are not without complications, particularly in the foot region, where achieving not only coverage but also mechanical function recovery is crucial ([3], Cho). While these approaches remain valuable, recent advancements suggest alternative strategies that minimize the need for premature implant removal and complex reconstructive procedures (Cho, [4]). In cases of acute infection, local debridement combined with antibiotic therapy is a viable option, provided the implant remains stable [5]. This approach allows fracture healing while adequately controlling infection through systemic antibiotics and rigorous debridement protocols. Even plate exposure seems not to be a contraindication for this treatment approach. As recent studies have shown that advanced local wound care, including negative wound pressure therapy and skin substitutes, can effectively manage plate exposure and often avoid the need for surgical flaps [2]. In selected cases as children or due to lack of alternative treatment strategies, conservative treatment with or without antibiotics and delayed implant removal has led to successful outcomes [2].

Therefore, we believe that exploring alternative treatment methods deserves attention. The present study aims to describe our experience in managing seven patients with plate exposure in the foot and ankle region. Employing a conservative approach that combines local wound care with advanced dressings, antibiotic treatment if the wound was previous infected and delayed implant removal in radiographic confirmed union. By sharing these outcomes, we aim to contribute to the growing body of evidence supporting alternative management strategies for plate exposure in orthopedic trauma patients.

## Materials and methods

This case series reports on seven consecutive patients treated for plate exposure at our institution from 2020 to 2025. The plate exposure was in four out of seven cases due to an acute infection and in the other cases no active infection was noticed before or after plate exposure. The cohort included all fracture of the foot and ankle:

- Four patients with malleolar fractures
- Two patients with calcaneal fractures
- One patient with a Lisfranc injury

### Case laboratory and inclusion criteria

Between 2020 and 2025, patients treated at our institution for postoperative wound complications following foot and ankle ORIF were retrospectively screened. During this period, several cases of wound dehiscence were observed; however, only patients with true exposure of the osteosynthesis hardware, defined as direct visualisation of the fixation plate, were considered eligible for inclusion. Patients presenting with wound dehiscence without direct hardware exposure were not included in this case series. All consecutive patients meeting this inclusion criterion were included.

### Patient characteristics

Among the seven patients analyzed, there were four men and three women with a medium age of 54 years (range: 20–77 years). Of these, only two patients were smokers, none reported drug use, and none had diabetes. The mean size of the plate exposure was 2.9 cm^2^, with a minimum of 0.4 cm^2^ and a maximum of 7 cm^2^.

All patients undergoing the index ORIF received standardized perioperative antibiotic prophylaxis with cefazolin (1.5 g) at induction, unless contraindicated due to allergy.

### Treatment protocol

In our case series, four patients developed an acute infection following open reduction and internal fixation for a fracture around the ankle. Infection was defined by both systemic symptoms and clinical signs of local infection, such as purulent drainage or erysipelas. The wound was surgically debrided and antibiotic therapy was initiated based on microbiological findings. The difficult wound healing after debridement resulted in plate exposure, which was managed with various wound care approaches in the outpatient setting (see Table 1).

**Table 1:** Patient characteristics, type of fracture, treatment strategies, antibiotic therapy, microbiological findings, and duration of plate exposure in the case series

Age	Fracture type	Surgical treatment	Time of plate exposure	Wound treatment during plate exposure	Antibiotic therapy	Biopsy	Plate exposure duration
77	Lateral Malleolar	ORIF, implant removal at 6 weeks	After ORIF	VAC Therapy	Co-Amoxicillin Until implant removal	Hardware removal: negative (on antibiotics)	5 weeks
69	Distal tibia (spiral) + fibula	Multiple surgeries, flap failure, delayed implant removal a year after initial trauma	After flap failure	NuShield	None	Implant exchange: S. epidermidis (1/4); hardware removal: negative, negative sonication	9 months after flap failure
55	Lisfranc	ORIF, implant removal at 3 months	After ORIF	VAC Therapy Apligraf	None	Implant removal: negative	3 months
21	Bimalleolar	ORIF, Debridement + VAC at 2 weeks, implant removal at 3 month	After debridement	NuShield	4 weeks Bactrim	Debridement: S. epidermidis resistant, Hardware removal: negative, histophathology negativ,	2 months
78	Calcaneus + Talus	ORIF, debridement partial implant removal (2 screws) at 2 weeks	After debridement	Apligraf + VAC Therapy	3 months Clindamycin	First debridement S. epidermidis, than negative (on antibiotics)	4 months
64	Trimalleolar	ORIF, implant change after 2 weeks, debridement + VAC after 3 weeks	After debridement	NuShield	3 months Ciprofloxacin	Debridement: E. cloacae, E. hormaechei	4 months
60	Calcaneus	ORIF, hardware removal at 9 months	After ORIF	Honey + standard dressing	None	Hardware removal: Negative, histopathology negative, sonication negative	3 months

In cases where plate exposure occurred without any clinical signs of infection, the wounds were treated in an outpatient setting. We recommend hardware removal after fracture consolidation for all patients; however, two patients either refused or delayed this procedure (see Table 1).

Of the seven patients, the four patients who had a local infection before plate exposure were treated with antibiotics until fracture consolidation. The antibiotic treatment lasted for a minimum of six weeks or until the plate was removed.

### Wound care management

- Standard Dressing Changes: These were performed initially during the inpatient stay and subsequently in the outpatient wound care clinic. The patient was seen in a one to two week interval after discharge. The choice of wound care technique was based on a pragmatic clinical assessment performed jointly by the orthopedic and wound care teams. The decision was guided by three main factors: (1) absence or control of infection, assessed clinically and, when indicated, by laboratory parameters; (2) stability of the fixation and need to preserve hardware until fracture consolidation; and (3) local wound characteristics, including size of exposure, level of exudate, presence of granulation tissue, and soft-tissue quality. Standard dressings were used for small, superficial wounds with favorable progression, negative pressure wound therapy was preferred in the presence of significant exudate or insufficient granulation tissue, and biological skin substitutes were applied once a clean and vascularized wound bed was achieved to promote epithelialization. In selected cases, medical-grade honey dressing was used as an adjunctive local treatment, particularly for superficial wounds requiring antimicrobial support and moisture control. The indication and use of honey dressing are detailed on a per-case basis in Table 1.
- Negative wound pressure therapy (NWPT): For the two patients managed with NWPT, dressing changes were conducted every five days in the outpatient setting.

### Wound dressing materials

For bedside dressing changes, two advanced wound care products were utilized:

- Apligraf^®^ (Organogenesis Inc., Canton, MA, USA): A novel bi-layered living skin equivalent. Apligraf is derived from foreskin fibroblasts combined with bovine type I collagen to create a neodermis over which cultured neonatal keratinocytes are placed. These keratinocytes proliferate, differentiate, and contribute to effective wound healing.
- NuShield^®^ (Organogenesis Inc., Canton, MA, USA): A dehydrated human placental allograft that retains all native layers of placental tissue, including the amnion, chorion, and spongy layer. This product is designed to enhance the wound healing process by preserving critical biological components.

### Follow-up protocol

Radiographic follow-up was performed to assess bone healing at the following intervals:

- 6 weeks
- 3 months
- 6 months
- 1 year

Bone consolidation was assessed through standard radiographs. When results were inconclusive, CT scans were conducted at the 3-month follow-up.

## Results

**Fig. 1:**
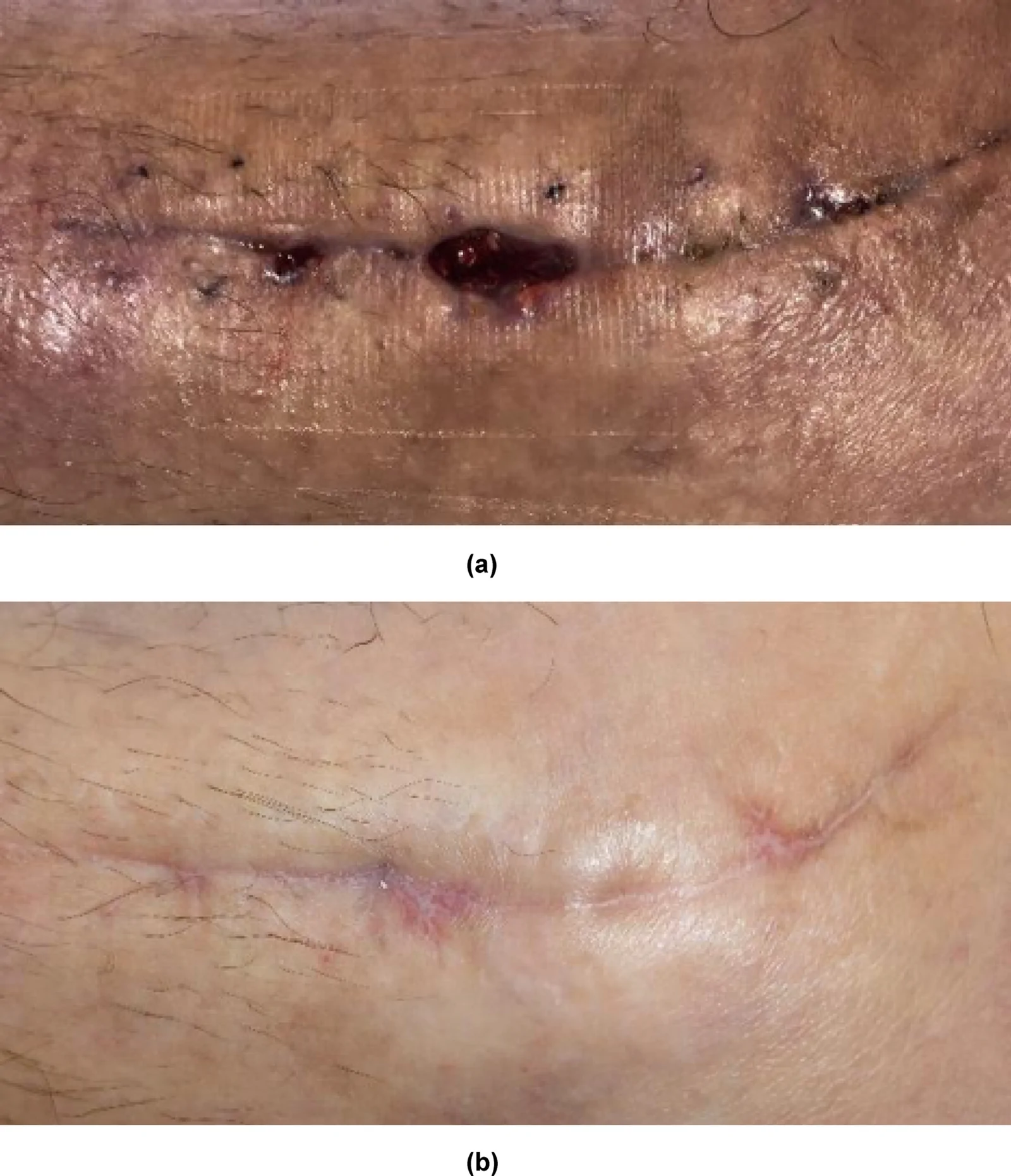
Example of a wound with the osteosythesis plate visibly before (**a**) and after (**b**) several months of extensive wound treatment

All seven patients achieved satisfactory bone healing without signs of nonunion, except for one patient who developed a nonunion of the medial malleolus at the one-year follow-up. In this case, the lateral malleolus and fibula had completely healed, and the patient was asymptomatic; therefore, no treatment was undertaken (Fig. 1).

The mean time to complete wound closure was 143 days (range: 85–243 days). Wound dressings using skin substitutes (Apligraf/NuShield) were maintained until full wound healing was achieved.

Plate removal was performed in five of the seven patients in average 169 days (range: 45–311 days) after the initial surgery. Out of the seven patients, only four received antibiotic therapy, with a mean duration of 83 days. This antibiotic treatment was due to an infection which leads to plate exposure.

At the time of hardware removal, bacteriological samples were consistently collected to assess for potential infection, and all results showed no bacterial growth. When histopathological examination or sonication was performed, these were also negative (Table 1).

## Discussion

Orthopedic implant exposure has two challenges which are frequently discussed in the literature, the challenge of wound closure and the risk of infection or contamination of the implant. Our conservative treatment approach for plate exposure demonstrated effectiveness, with all patients achieving good clinical outcomes, wound closure by secondary wound healing and bone healing. The protocol, which included the use of antibiotic therapy where needed combined with advanced dressings, eliminated the need for invasive interventions such as extended flaps coverage.

This method aligns with previously reported case reports. For instance, Melhem et al. reported successful outcomes in three pediatric patients with extensive plate exposure treated conservatively with wound care, antibiotic therapy, and delayed implant removal after confirmed bone healing [2]. In a case reported by Lee et al., a patient with an exposed titanium cranioplasty plate declined the recommended treatment strategy. Instead, the patient was successfully managed with a local application of platelet-rich plasma and a dermal autograft, which was considered a scaffold to support cellular migration [6]. A similar approach was described by Lee et al. in another report involving 14 patients with exposed orthopedic implants. Initial treatment included the use of MatriDerm—a dermal collagen-elastin matrix—combined with negative pressure wound therapy. Once adequate granulation tissue had formed, a split-thickness skin graft was applied. All patients experienced successful wound healing without reported complications [7]. In contrast to this study, our patients were treated with outpatient open wound treatment and did not require an additional surgery.

Similarly, the use of advanced wound management techniques, such as negative pressure wound therapy and skin substitutes, has gained prominence as effective strategies to promote tissue granulation and minimize the need for surgical flap coverage [8]. Kunze et al. mentioned that negative pressure wound therapy (NPWT) can enhance tissue granulation, reduce bacterial burden, and promote closure of wounds [9]. One of the key aspects of our protocol was the use of advanced dressings like Apligraf^®^ and NuShield^®^ to facilitate wound healing. These skin substitutes were successfully utilized, as demonstrated by several studies, including those by Metsemakers et al. and Masters et al., who highlighted the positive role of biological materials in promoting wound healing and reducing postoperative complications [10, 11].

The issue of implant infection following exposure remains a topic of ongoing discussion. This is particularly noteworthy in our case series, as we achieved successful skin closure over the exposed plates. Moreover, in the instances where the plates were subsequently removed, microbiological analysis revealed no presence of pathogens.

In our case series we have several cases with a acute infection which lead to plate exposure and was successfully treated with systemic antibiotics. This approach aligns with findings in literature, where implant retention has been successfully achieved in many cases, even in the presence of infection, due to modern techniques such as local and systemic antibiotic treatment. Numerous studies support the idea that a conservative approach with delayed plate removal can be a valid alternative to traditional methods of early removal of the fixation hardware. The approach of conservative treatment, even with clinical sign of infection, as shown by Tsang et al. and Casiraghi et al., suggests that implant retention is feasible in patients with acute infections when treated promptly with surgical washouts, targeted antibiotic therapy, and debridement [12, 13]. Our study also confirms the findings by Depypere et al. and Onsea et al., who emphasize that a conservative approach, monitored through regular radiographic follow-ups, allows for bone healing without the necessity of early plate removal [4, 14].

This conservative approach has led to reduced morbidity compared to traditional approaches, avoiding the complications typically associated with invasive treatments, such as early plate removal or flap coverage. Our findings align with the recommendations from Metsemakers et al., who emphasize the importance of a multidisciplinary treatment approach and the use of antibiotic therapies to prevent complications and improve clinical outcomes [10].

Moreover, Depypere et al. emphasized the importance of a multidisciplinary approach to managing fracture-related infections, underlining that antibiotic suppression combined with meticulous wound care and delayed hardware removal may improve outcomes in select patients [4].

This study highlights that plate exposure alone is not an indication for antibiotic therapy or acute surgical treatment. In the absence of infection criteria such as redness, warmth, swelling, or frank pus, or signs of systemic infection in the laboratory results a conservative treatment approach with bedside dressing changes followed by outpatient wound management can be successfully adopted. This approach does not compromise fracture healing.

It should be acknowledged that advanced wound care strategies may be associated with relevant costs. However, we believe that the overall burden of care should be interpreted in comparison with the current gold standard, namely early flap coverage, which is more invasive for the patient and not necessarily less costly when considering surgical complexity, hospitalization, and potential donor-site morbidity.

Given the small sample size and descriptive nature of this case series, no formal institutional protocol changes were implemented; however, this experience supports consideration of a conservative management pathway in carefully selected patients.

Our results suggest that a less aggressive treatment approach can be adopted until bone consolidation is achieved, thus avoiding additional surgical stress and the risk of complications in an already fragile and compromised area. Based on our observations, we hypothesize that distinguishing between contamination and infection is crucial. An exposed plate might lead to bacterial contamination, which does not necessarily lead to an infection requiring acute surgical debridement and plate removal [15, 16].

### Limitations

This study has several limitations. First, the retrospective design and the small sample size limit the generalizability of the findings. Additionally, the patient population was heterogeneous in terms of fracture type, anatomical location, and treatment course, which introduces variability in outcomes and makes direct comparisons challenging. Another important limitation is the lack of systematic microbiological or histopathological assessment in all cases. While bacteriological samples were collected at the time of implant removal, no biopsies were performed during the initial management. Therefore, the presence of biopsy-negative infections cannot be entirely excluded. Residual or subclinical infection after fracture healing and wound closure was not specifically investigated with a dedicated post-operative surveillance protocol. However, since both fracture union and complete wound healing were achieved in all cases, the primary outcome of this study was reached, and the presence of residual infection within the removed hardware was not considered a primary endpoint of this analysis. This factor should be considered when interpreting the results, as subclinical or low-grade infections might have gone undetected despite the apparent clinical and radiographic resolution.

No historical or concurrent comparison group was available, and therefore the findings of this case series should be interpreted as descriptive and hypothesis-generating rather than comparative.

## Conclusion

This case series demonstrates that a conservative approach to managing plate exposure, combining antibiotic therapy if needed, advanced wound care with skin substitutes, and delayed plate removal, can be highly effective in achieving bone healing without the need for early implant removal or flap coverage.

The results of this study contribute to the growing body of evidence supporting implant retention as a viable option in the management of plate exposure, particularly in patients without signs of active infection or with well-controlled infection. The use of advanced wound care techniques, such as NWPT and biological skin substitutes, plays a key role in promoting wound healing and preventing complications.

## Data Availability

The datasets generated and/or analyzed during the current study were collected using REDCap electronic data capture tools hosted at [anonymised]. Data are available from the corresponding author on reasonable request.
